# *Toxoplasma gondii* Seropositivity and Co-Infection with TORCH Pathogens in High-Risk Patients from Qatar

**DOI:** 10.4269/ajtmh.2010.09-0530

**Published:** 2010-04

**Authors:** Marawan A. Abu-Madi, Jerzy M. Behnke, Haydee A. Dabritz

**Affiliations:** College of Arts and Sciences, Department of Health Sciences, Qatar University, Doha, Qatar; School of Biology, University of Nottingham, Nottingham, United Kingdom; Infant Botulism Treatment and Prevention Program, California Department of Public Health, Richmond, California

## Abstract

Testing of patients who are deemed to be at high risk for TORCH pathogens, e.g., pregnant women, their fetuses, neonates, and acquired immunodeficiency syndrome (AIDS) patients, is important so that specific treatment can be initiated. This study included 1,857 such patients between 2005 and 2008. Logistic regression was used to evaluate factors associated with *Toxoplasma gondii* seropositivity. Among 823 women of childbearing age, 35.1% and 5.2% tested positive for *T. gondii* IgG and IgM, respectively. Three infants ≤ 6 months of age (0.8% of 353) were congenitally infected. Factors associated with *T. gondii* IgG seropositivity included older age, East Mediterranean or African nationality, positive cytomegalovirus (CMV) and herpes simplex virus (HSV)-1 serostatus, and negative rubella IgG results. The decreasing prevalence of IgM antibodies between 2005 and 2008 suggested that exposure to *T. gondii* from food or environmental sources declined over this period in Qatar. Population-based studies of newborns would be helpful to accurately estimate incidence of congenital toxoplasmosis.

## Introduction

*Toxoplasma gondii* is a ubiquitous parasite whose definitive hosts are members of the Felidae (cat family). Cats shed millions of environmentally resistant oocysts in their feces after primary infection and are usually without clinical manifestations of disease.^1^–^4^ Intermediate hosts include almost all warm-blooded mammals and birds, including humans, who accumulate infectious, quiescent stages (bradyzoites) of the parasite in their tissues, particularly in skeletal muscle and the brain.^5^,^6^ Intermediate hosts may acquire infection by consuming raw or undercooked flesh from other intermediate hosts,^5^ or by ingesting oocysts from the environment.^7^,^8^ Environmental sources of *T. gondii* (oocysts) include soil, water, shellfish, fruits, and vegetables.^9^–^16^

*Toxoplasma gondii* is of particular concern in humans because of the potential for transmitting the disease to the unborn fetus if the mother is infected for the first time during pregnancy.^17^,^18^ Toxoplasmosis most commonly manifests as a mild, flu-like illness with low-grade fever, myalgia, malaise, and headache, but primary infection in humans may also cause spontaneous abortion, fetal mental and pyschomotor retardation, retinochoroiditis, encephalitis, and hepatitis.^6^,^17^,^18^ Patients with a history of recent miscarriage, ocular infection, jaundice, hepatosplenomegaly, and cirrhosis of the liver may be referred into a testing protocol termed “TORCH” (*T. gondii*, other [if done, e.g., syphilis, varicella zoster virus, human immunodeficiency virus, and parvovirus B19], rubella, cytomegalovirus [CMV], and herpes simplex viruses [HSVs]), to rule out infections with similar clinical presentations.

The TORCH infections can cause serious illness or death to the fetus or neonate, so TORCH testing is important to protect the health of neonates that may have been exposed to one or more TORCH pathogens *in utero*. It is also important to identify the etiologic agent associated with clinical disease in symptomatic adults so that appropriate treatment can be initiated. The classic triad of symptoms associated with congenitally acquired toxoplasmosis includes hydrocephaly, intracranial calcifications, and ocular lesions.^17^ Rubella infection of the fetus causes birth defects and blindness, hearing loss, and mental retardation.^19^ Congenital CMV has similar manifestations to toxoplasmosis and rubella that include sensorineural hearing loss, mental retardation, and retinochoroiditis.^20^ Neonatal HSV (usually acquired during vaginal delivery) may lead to external infection of the skin, eyes, and mouth, central nervous system (CNS) infection (encephalitis), or disseminated infection involving several organs such as the brain, liver, and lung.^21^ Disseminated infection is a more frequent cause of mortality.^21^ Most clinical disease in neonates is caused by HSV type 2 (HSV-2) infection.^21^,^22^

In Qatar, better prenatal care and greater vigilance with regard to TORCH infections may lead to earlier and more effective therapy. No specific *T. gondii* prevention program exists in Qatar other than routine prenatal counseling, which may or may not include advice about how to prevent toxoplasmosis. Increasing concern has been raised about toxoplasmosis because of the large, indigenous feral cat population in the capital city of Doha, where most of the inhabitants live. Doha and its surrounding communities had experienced problems controlling rodents for decades, so domestic cats were introduced in the 1960s to ameliorate the problem. The cat population is now estimated to exceed 2 million animals (Cat Control Unit, personal communication). Cats are rarely kept as pets in Qatar and the vast majority leads a feral existence on the streets, congregating near human dwellings, businesses, restaurants, and in the market places where food for human consumption is prepared and traded.^23^ They survive by scavenging on garbage and preying on rodents, and some are supplemented with food by the local residents.

This analysis updates and extends an earlier study of patients referred for TORCH testing in Qatar.^24^ The aims were to determine the prevalence of *T. gondii* in the subpopulations of women of childbearing age and infants from among all patients referred for TORCH testing in Qatar, and to determine if specific TORCH pathogens (CMV, HSV-1, HSV-2, and rubella) were associated with *T. gondii* infection in patients referred for TORCH testing, after controlling for demographic *T. gondii* risk factors.

## Materials and Methods

### Study location, selection of subjects, and inclusion criteria.

Doha, the capital city of Qatar, is located on the Arabian Gulf and encompasses about 285 km^2^. The city is populated by about 1 million residents, many of whom are immigrants from other Middle Eastern countries, Africa, and Asia. The climate of Qatar is arid, with sparse annual rainfall averaging only 0.1–3.2 cm.

The entire patient population included persons who had symptoms compatible with those of TORCH pathogens (ocular disease, hepatosplenomegaly, cirrhosis), women with a history of miscarriage(s), and their most recent child, who was usually < 1 year of age. Testing was carried out in Qatari hospitals and outpatient clinics between 2005 and 2008, and patients came from such specialties as maternity, pediatrics, internal medicine, and gastroenterology. Patient confidentiality was maintained throughout and the data set were de-identified so as to mask patient identity from the investigators. The study was approved by the Medical Research Center & Research Committee at Hamad Medical Cooperation, Qatar (research protocol no. 8036/08).

### Blood collection and serological tests.

Each subject had 5 mL of whole blood collected by venipuncture in plain tubes. Blood samples were then transported to the virology laboratory at Hamad Medical Corporation according to hospital arrangements, centrifuged to remove blood cells, and stored at +4°C for 48 hours or frozen at −20°C for longer storage. Serologic tests for anti-*T. gondii* IgG and IgM antibodies were performed as previously described.^24^ Commercially available enzyme immunoassay Enzygnost kits (Dade Behring GmbH, Marburg, Germany) were used to detect the presence of antibodies against *T. gondii*, rubella virus, and CMV, and the Novagnost EIA kits (NovaTec Immundiagnostica GmbH, Dietzenbach, Germany) for HSV-1/HSV-2. Sera with values of < 10 IU/mL were defined as negative for rubella IgG antibodies and those with values ≥ 10 IU/mL as positive. The latter test has a published sensitivity (Se) of 100% and specificity (Sp) 98.5%. Rubella IgM testing was not included in this analysis because the testing protocol for rubella IgM included only patients with negative rubella IgG results and symptoms of fever and rash, which increases the likelihood of the patient being a clinical rubella case. For CMV, sera with values < 0.5 IU/mL were defined as negative, those between 0.5 and 0.7 IU/mL as equivocal, and those > 0.7 IU/mL as positive. The reported Se and Sp of this test was 99.3% and 98.2%, respectively. For CMV IgM, sera were considered negative if the ratio of the optical densities, OD_sample_/OD_cutoff_, were < 90%, equivocal between 90% and 100% and positive if > 100%, and the manufacturer reported Se and Sp of 95% and 100%, respectively. In HSV-1 and HSV-2 Novagnost tests, sera were considered negative for HSV-1 or HSV-2 IgM or IgG antibodies if values were < 8.5 IU/mL, equivocal between 8.5 and 11.5 IU/mL, and positive if > 11.5 IU/mL. Se and Sp, respectively, for each of the four tests were reported as follows: HSV-1 IgG and IgM > 95% and > 95%, HSV-2 IgG 87.5% and 94.1%, and HSV-2 IgM > 95% and > 95%. The HSV-1 and HSV-2 immunoassays are considered to be semi-quantitative.

### Definition of variables.

Age was classified into ranges by yr as previously described,^24^ except that infants were classified into ages of ≤ 6 mo and 7–< 12 mo. In this analysis, the age group of 7–< 12 mo was used as the referent group because about one-quarter of infants ≤ 6 mo of age had IgG antibodies against *T. gondii* in their sera that were most likely acquired *in utero*. Maternally acquired antibodies usually disappear by 6 mo of age.^25^ However, IgG antibodies in infant sera can also appear as a result of congenital infection.^26^ For patients ≥ 12 mo of age, the categories in yr were 1–< 2, 2–10, 11–20, 21–29, 30–45, and > 45. Presence of IgM antibodies in infant sera suggests that toxoplasmosis was transmitted congenitally,^27^,^28^ but the tests lack sensitivity, missing 50–75% of incident cases.^27^,^29^,^30^ Prevalence of *T. gondii* antibodies in the female population was also assessed separately by age in yr to determine *T. gondii* prevalence in females of childbearing age (15–45 yr).

Subjects in the study came from 55 countries that were grouped into the following geographic regions: Arabian Peninsula (Bahrain, Iraq, Kuwait, Oman, Qatar, Saudi Arabia, United Arab Emirates, and Yemen); Africa (Algeria, Egypt, Eritrea, Ethiopia, Ivory Coast, Kenya, Libya, Mauritania, Morocco, Senegal, Somalia, Sudan, and Tunisia); Asia (Afghanistan, Bangladesh, Iran, India, Indonesia, Macao, Malaysia, Nepal, North Korea, Pakistan, Philippines, Singapore, South Korea, Sri Lanka, and Vietnam); Eastern Mediterranean/Eastern Europe (Armenia, Bulgaria, Jordan, Lebanon, Palestine, Romania, Syria, and Turkey); American continent (Canada, Cuba, United States, and Venezuela); and Other/Unknown (Australia, France, New Zealand, Spain, Russia, Ukraine, and United Kingdom). Year of testing was included as a categorical variable with 2005 as the referent year. Association of *T. gondii* seropositivity with TORCH pathogens included rubella (IgG) status, CMV IgG/IgM status, HSV-1 IgG/IgM status, and HSV-2 IgG/IgM status.

### Statistical analyses.

Estimates for the incidence of congenital toxoplasmosis were determined using two methods: defining a case as an infant ≤ 6 mo of age with concurrent IgM and IgG *T. gondii* antibodies in serum; or a woman of childbearing age with IgM antibodies to *T. gondii* in her serum. Both methods used the total number of live births in Qatar between 2005 and 2008 as the denominator for population size and assumed that all cases of congenital toxoplasmosis or incident cases of toxoplasmosis in pregnancy would have been detected. For the estimate based on women of childbearing age with *T. gondii* IgM antibodies, the probability of *T. gondii* transmission to the fetus was assumed to be between 25% and 50%. The lower limit of the 95% confidence interval (CI) for the estimate based on 25% transmission probability and the upper limit of the 95% CI for the estimate based on 50% transmission probability delimited the ranges for the estimates derived for IgM-positive women of childbearing age. Confidence intervals were calculated using Szklo and Nieto, Appendix A.^31^

The binary logistic regression analyses were conducted in Minitab Release 14.2 (Minitab Inc., State College, PA). Each explanatory factor in turn was fitted alone (one-way analyses) with the dependent variable (presence/absence of antibodies to *T. gondii*) and then full factorial models were simplified by using stepwise backward elimination. Factors were then fitted in minimum sufficient models where *P* values were considered significant if ≤ 0.05. The Hosmer-Lemeshow test was used to verify model fit. The Cochran-Armitage test was used to evaluate a trend of increasing *T. gondii* IgG seroprevalence with age and for decreasing IgM seroprevalence from 2005 to 2008 using SAS version 9.2 (SAS Institute Inc., Cary, NC).

## Results

**Figure 1.**
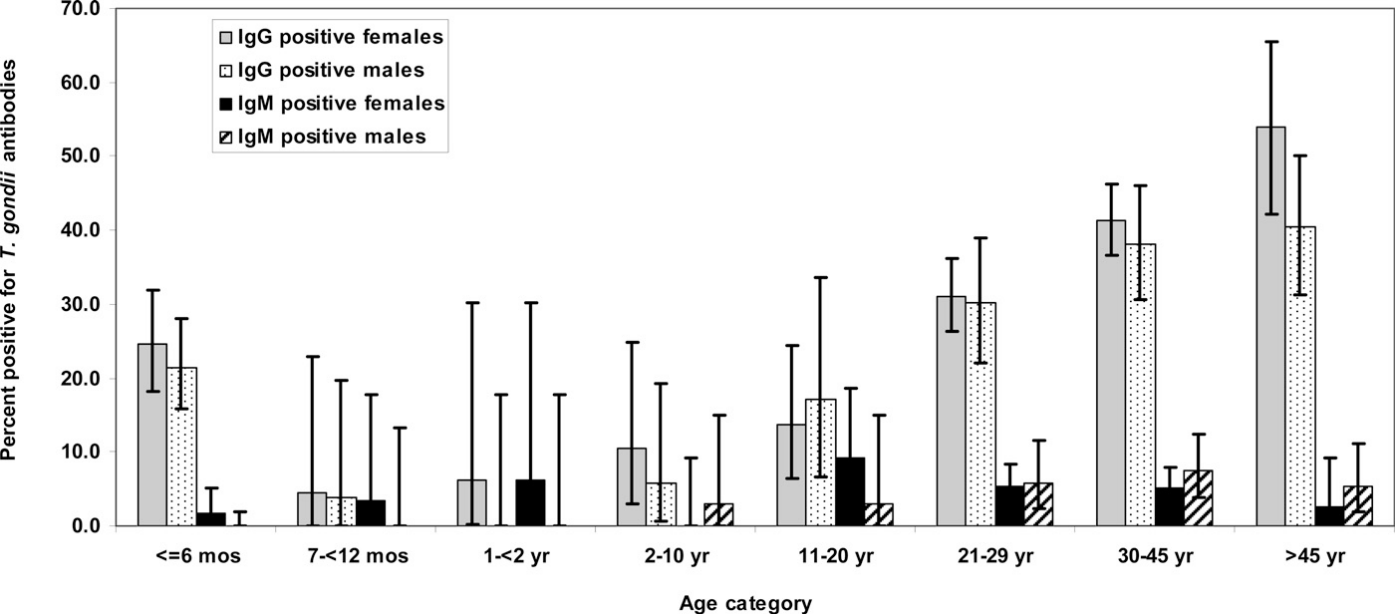
*Toxoplasma gondii* IgG and IgM seroprevalence by age category and gender in high-risk patients referred for TORCH testing in Qatar between 2005 and 2008. Exact binomial 95% confidence intervals (CIs) were computed using SAS software (SAS Institute Inc.).

Of the 1,857 patients referred for TORCH testing in 2005–2008, 4.1% percent (*N* = 3 missing data) and 30.8% of patients tested positive for IgM and IgG *T. gondii* antibodies, respectively (Table 1). *Toxoplasma gondii* IgG and IgM seroprevalence by age group for residents of Qatar is depicted in Figure 1. Among 823 women of childbearing age (15–45 yr), 289 (35.1%) had IgG antibodies and 43 (5.2%) had IgM antibodies against *T. gondii* (*N* = 1 of unknown IgM status). *Toxoplasma gondii* IgG seroprevalence increased with advancing age in both males and females, with a combined prevalence of 8% in 2 to 10 years of age compared with 54% in patients over 45 years of age. The Cochran-Armitage trend test by age category showed a significant association with increasing age (Z-statistic 7.97, 7 degrees of freedom [df], *P* < 0.01). This was markedly different from the age prevalence of rubella virus and CMV. Rubella IgG seroprevalence ranged from 50% to 78% in all age groups from 1–< 2 yr and up (Table 1). In Qatar, vaccination against rubella has been mandatory for children before their first birthday since the 1970s. The CMV IgG seroprevalence was around 80% at young ages (2 to 10 years of age) and generally > 90% in all age groups from 11–20 yr and up. In contrast, HSV-1 and HSV-2 IgG seroprevalence almost doubled in the age group encompassing teens (11–20 yr) compared with 2 to 10 years of age, and remained relatively stable thereafter. Notably, prevalence of HSV-1 IgG was higher than that of HSV-2 in adults (21 and up): 65–79% compared with 17–30%, respectively.

Of the 353 infants in the study aged ≤ 6 mo, 81 (22.9%) had detectable anti-*T. gondii* IgG antibodies that may be passively transferred by the placenta, compared with only 3 (0.8%) with IgM antibodies (Table 1). All three infants with IgM titers also tested positive for IgG antibodies and were most likely congenitally infected. Using cases as defined for infants and total live births of 13,401 in 2005, 14,120 in 2006, 15,681 in 2007, and 17,210 in 2008, the annual incidence of congenital toxoplasmosis from 2005 to 2008 was estimated to be 0.5 cases (95% CI 0.1–1.5 cases) per 10,000 live births. If the number of cases was based on the detection of anti-*T. gondii* IgM antibodies in high-risk women of childbearing age with the parameters defined in the methods section, the estimated incidence of congenital toxoplasmosis between 2005 and 2008 ranged from 0.9–5.5 cases per 10,000 live births.

In the one-way analyses, presence of *T. gondii* IgG antibodies was positively associated with older age; African, Asian, or East Mediterranean/European nationality; positive CMV IgG status; positive HSV-1 IgG status; and positive HSV-2 IgM status (Table 2). It was negatively associated with male gender and rubella IgG seropositivity. In contrast, there was no association between *T. gondii* IgM status and age or nationality, but positive tests were associated with seropositivity to HSV-1 IgG and HSV-2 IgM antibodies. In addition, the one-way analysis revealed a lower prevalence of positive *T. gondii* IgM results in 2008 compared with 2005 (Table 2). A subsequent trend test indicated a decline in the prevalence of anti-*T. gondii* IgM antibodies between 2005 and 2008 (Z-statistic 2.09, 1 df, *P* = 0.037).

In multifactorial analyses (Table 3), anti-*T. gondii* IgG seropositivity was associated with older age (χ^2^ 78.43, 7 df, *P* < 0.001); African, East Mediterranean, or East European nationality (χ^2^ 42.92, 5 df, *P* < 0.001); positive tests for CMV IgG (Z-statistic 1.93, 1 df, *P* = 0.054), and HSV-1 IgG (Z-statistic 2.40, 1 df, *P* = 0.016); and negative rubella IgG serostatus (Z-statistic −4.53, 1 df, *P* < 0.001). No interactions were significant, and neither gender nor the HSV-2 IgM response came into the equation. Anti-*T. gondii* IgM seropositivity was negatively associated with year of testing (lower in 2008 versus 2005) and positively associated with the presence of HSV-2 IgG antibodies (Table 4). The positive association with the HSV-1 IgG detected in the one-way analyses was not confirmed by the multifactorial analysis.

## Discussion

The results of TORCH testing suggest that approximately 65% of women of childbearing age in Qatar have no IgG antibodies to *T. gondii* (35.1% were IgG positive) and hence are susceptible to infection with toxoplasmosis. Pregnant women should take appropriate precautions to protect themselves against infection. Such precautions include cooking meat, especially lamb and pork, until it is well done; thorough washing of cutting boards used to prepare meat; wearing gloves when gardening; rigorous hand washing after handling raw meat or working in the soil; and avoiding contact with cat feces.^32^ Pregnant women who must clean out cat litter boxes should wear disposable gloves and collect feces daily, because it takes about 24 hr for oocysts to sporulate and become infectious at room temperature.^33^ Forty-three women of childbearing age (5.2%) tested positive for anti-*T. gondii* IgM antibody. If they were pregnant, IgG avidity testing is the preferred method to confirm recent infection,^34^ because IgM antibodies can persist for months after initial infection in some individuals.^35^,^36^ Alternate testing algorithms use higher IgM serologic titers as good indicators of acute infection when economic considerations preempt additional tests.^37^,^38^

The presence of IgM antibody in three infants ≤ 6 mo of age suggested that they were congenitally infected. However, IgM testing reportedly fails to detect 50–75% of infected infants; therefore, 6–12 cases of congenital toxoplasmosis may have occurred between 2005 and 2008.^29^,^30^,^39^ The results of IgM testing in women of childbearing age with high-risk pregnancies (history of miscarriage) support this hypothesis, because 43 cases of toxoplasmosis acquired during pregnancy would be expected to result in 11–21 cases of congenital toxoplasmosis, assuming a 25–50% probability of transmission to the fetus *in utero*. In north Jordan, women with a history of 3 to 7 miscarriages were twice as likely to test seropositive for *T. gondii* as women with normal pregnancies.^40^ Women without high-risk pregnancies are also at risk for acquiring toxoplasmosis and transmitting it to their fetus, but we do not know if the risk is similar to or differs from that of women with high-risk pregnancies in Qatar. Our estimates for the incidence of congenital toxoplasmosis in Qatar are therefore speculative and should be confirmed by population-based studies. In the absence of universal screening, primary prevention of congenital toxoplasmosis through heightened maternal education efforts can be an effective strategy to prevent transmission to the fetus.^41^,^42^

The overall *T. gondii* IgG seroprevalence (33%) during the time period (Table 1) was similar to cross-sectional studies from Bahrain (28%),^43^ Saudi Arabia (32%, 25%, 36%, and 29% reported in 1991, 2001, 2002, and 2006, respectively),^44^–^47^ and the United Arab Emirates (34%),^48^ but much lower than that reported in two studies from Kuwait (96% and 58%) in the 1980s.^49^,^50^ Overall, prevalence of *T. gondii* IgM antibodies (4%) was also similar to countries in the Arabian Peninsula, e.g., Saudi Arabia (5% and 5.6% in the eastern region and Makkah, respectively),^45^,^47^ and the United Arab Emirates (3%).^48^ Comparisons between the present and earlier studies should be made with caution, because different tests were used and most of the studies were conducted in specific subpopulations, such as pregnant women or women of childbearing age.

*Toxoplasma gondii* IgM prevalence in high-risk patients declined significantly between 2005 and 2008, but this was not the case for IgG seroprevalence. The latter is a measure of individuals infected months or years earlier; therefore, it is unlikely we would detect a decline until incidence decreases significantly for 5 to 10 years. Declining IgM seroprevalence suggests that knowledge about how to prevent *T. gondii* infection may have improved, the prevalence of *T. gondii* cysts in food animals in the region is declining, or that the feral cat control program is reducing the quantity of oocysts entering the environment. According to the Ministry of Public Health, there have been no changes in public education programs with regard to *T. gondii*. The prevalence of *T. gondii* in meat, most likely sheep and goats, imported into Qatar for human consumption is unknown. Reports from other countries in the region since 2001 found *T. gondii* prevalence of 23% to 52% in sheep,^46^,^51^–^56^ suggesting that lamb, if it is undercooked (a prevalent cultural practice in this area), could be a source of *T. gondii* infection. Since the feral cat control program began in 2006, 9,637 cats have been spayed or neutered and 282 cats of 871 tested (32.3%) were serologically positive for *T. gondii* antibodies. It seems likely that the cat control program may be reducing environmental exposure to *T. gondii* oocysts by controlling reproduction and lessening the number of susceptible kittens entering the population, but testing of residents living within and outside trapping areas would be required to verify this hypothesis.

Multifactorial analysis found that the presence of *T. gondii* IgG antibodies was positively related to age, African or East Mediterranean/East European nationality, and the presence of CMV IgG and HSV-1 IgG antibodies, whereas it was negatively associated with rubella IgG seropositivity. Increasing *T. gondii* seroprevalence with age has been well documented, suggesting that exposure is constant over the life span.^24^,^57^,^58^ Persons originating from African nations, the East Mediterranean, and Eastern Europe were 2.3 times more likely to have *T. gondii* IgG antibodies than persons who came from countries in the Arabian Peninsula. The increased risk may reflect cultural differences in eating practices, such as consumption of rare or undercooked meat and choice of meat, and weather patterns that promote oocyst survival. Uduman and others^48^ also found that *T. gondii* seroprevalence in Mediterranean Arabs was about twice as high as that of persons from the Arabian Gulf countries or the United Arab Emirates. Consumption of rare lamb or goat is common in the Eastern Mediterranean, Turkey, and Iran.^41^,^59^,^60^ Extremely high temperatures and prolonged periods without rainfall in the Arabian Peninsula may inactivate oocysts in the environment rapidly, thereby reducing transmission to humans directly by soil contact or indirectly by food animals such as sheep and goats. For example, oocysts can survive 32 d at 35°C but only 9 d at 40°C.^33^ Minimum and maximum temperatures in Qatar range from 25 to 45°C, respectively, from June to September and rarely dip below 10°C in the winter months, and it is unclear how long oocysts might survive in this desert climate. In Costa Rica, oocysts in soil survived for up to 1 yr even when air temperatures reached 30°C.^61^ Studies of oocyst survival in the native Qatari climate would help to determine if oocysts represent a health hazard to persons with soil contact or if specific seasons of the year pose a greater hazard to humans and animals for acquiring *T. gondii* from the environment.

Three other TORCH pathogens were associated with *T. gondii* IgG seropositivity in the present analysis. Adjusting for age and nationality, *T. gondii-*seropositive patients were 1.94 and 1.35 times more likely than seronegative patients to have previous exposure to CMV and HSV-1, respectively (Table 3). They were about half as likely as seronegative patients to have IgG antibodies to rubella virus (odds ratio [OR] = 0.59). The CMV is transmitted directly from person to person, particularly between children, in saliva, urine, and genital secretions.^62^ Most of the children and adolescents in Qatar appear to have been exposed to CMV with seroprevalences of 79% in the 2–10-year and 91% in the 11–20-year age groups. Poor socioeconomic conditions that are characterized by overcrowding and a lack of hand hygiene, and placing children in daycare facilities, promote CMV transmission.^63^ Low socioeconomic status is also associated with *T. gondii* infection,^57^,^64^–^66^ which may explain why persons with positive CMV serostatus were more likely to be seropositive for *T. gondii* IgG antibodies. The HSV-1 is an alpha-herpes virus that produces orolabial blisters or lesions. It remains latent for the life of the infected host and causes intermittent viral shedding, at which time it is infectious to susceptible persons. In the past, most primary HSV-1 infections were oral, and were acquired in childhood by direct mucosal or cutaneo-mucosal contact with an infected person.^67^ Improved hand hygiene in developed countries has reduced the incidence of oral HSV-1 in childhood, whereas primary genital HSV-1 (acquired during sexual contact) has become more common in the teen years and young adulthood.^68^–^71^ In Israel, genital HSV-1 has become more prevalent than HSV-2.^71^ The association of HSV-1 IgG antibodies with *T. gondii* IgG seropositivity may be related to poor socioeconomic conditions during childhood, because HSV-1 IgG was highly prevalent in the 2–10-year (34%) and 11–20-year (61%) age groups. Much like the association with CMV, an association between HSV-1 and socioeconomic factors has been documented in other countries.^69^,^72^,^73^

Unfortunately, vaccines for CMV and HSV-1 have not yet been developed, but a vaccine against rubella virus has been available since 1969.^74^ Most nations in the Middle East, including Qatar, have rubella in their national immunization schedules, whereas almost all nations in Africa do not.^75^ Because the rubella virus IgG antibody test does not discriminate between vaccine-induced and naturally acquired immunity, seropositivity to rubella IgG is likely to be a surrogate for vaccine status. The reverse association for rubella virus with *T. gondii* IgG seropositivity is probably related to higher socioeconomic status, which entails better access to health care, more hygienic living conditions, and less occupational contact with soil. Soil contact, either by occupational exposure or gardening, is a well-recognized risk for *T. gondii* seropositivity in many nations.^41^,^57^,^76^–^79^ It would have been desirable to obtain information about soil exposure and other factors known to be associated with *T. gondii* seropositivity, such as cat contact and consumption of undercooked meat, from patients referred for TORCH testing, but these data were not collected. Such data could help determine the extent to which environmental exposure to oocysts and/or exposure by the food chain contribute to human toxoplasmosis in Qatar, so that appropriate preventive measures can be adopted.

Only limited conclusions can be drawn about the prevalence of *T. gondii* in Qatar from this study, because it focused on a specific, high-risk population. If patients included in the study were more likely to be infected with toxoplasmosis, we would then overestimate the prevalence of *T. gondii* in Qatar. Conversely, if we failed to detect asymptomatic cases, which are more likely, the burden of toxoplasmosis may be underestimated. Nonetheless, the proportion of previously infected persons was similar to other countries in the Arabian Peninsula. The high percentage of at-risk females in their childbearing years (65%) suggests that pregnant women should receive counseling during their antenatal visits on how to prevent *T. gondii* infection. Population-based studies or universal screening of newborns over a specified time period could elucidate the true burden of congenital toxoplasmosis in Qatar and suggest priorities for public health action. Future studies would benefit from questions that assess exposure to previously identified risk factors for *T. gondii* infection, such as recreational or occupational exposure to soil, drinking water source, contact with domestic pets and food animals, and practices related to cooking and preparing meat.

## Figures and Tables

**Table 1:** The number and percentage of high-risk patients testing positive for TORCH pathogens in Qatar by gender and age group, 2005–2008*

Age group	*Toxoplasma gondii* IgG n (%)	*T. gondii* IgM n (%)	Rubella IgG n (%)	CMV IgG n (%)	CMV IgM n (%)	HSV-1 IgG n (%)	HSV-1 IgM n (%)	HSV-2 IgG n (%)	HSV-2 IgM n (%)
Females									
≤ 6 mo (*N* = 167)	41 (25)	3 (2)	104 (62)	157 (95)	9 (5)	86 (52)	0 (0)	51 (31)	0 (0)
7–11 mo (*N* = 22)	1 (5)	0 (0)	3 (14)	16 (73)	1 (5)	3 (14)	0 (0)	0 (0)	1 (5)
1–< 2 yr (*N* = 16)	1 (6)	1 (6)	8 (50)	10 (62)	1 (6)	3 (19)	0 (0)	1 (6)	0 (0)
2–10 yr (*N* = 38)	4 (11)	0 (0)	25 (66)	30 (79)	3 (8)	13 (34)	1 (3)	5 (13)	5 (13)
11–20 yr (*N* = 66)	9 (14)	2 (3)	42 (64)	60 (91)	2 (3)	40 (61)	1 (2)	14 (21)	6 (9)
21–29 yr (*N* = 367)	114 (31)	20 (5)	285 (78)	352 (96)	11 (3)	246 (67)	6 (2)	87 (24)	27 (7)
30–45 yr (*N* = 404)	167 (41)	21 (5)	300 (74)	394 (98)	10 (2)	301 (75)	2 (0.5)	122 (30)	33 (8)
> 45 yr (*N* = 76)	41 (54)	2 (3)	49 (64)	74 (97)	2 (3)	60 (79)	0 (0)	14 (18)	4 (5)
**Total** (*N* = 1,156)	378 (33)	49 (4)	816 (71)	1093 (95)	39 (3)	752 (65)	10 (1)	294 (25)	76 (7)
Males									
≤ 6 mo (*N* = 186)	40 (22)	0 (0)	127 (68)	178 (96)	8 (4)	101 (54)	1 (1)	60 (32)	1 (1)
7–11 mo (*N* = 26)	1 (4)	0 (0)	2 (8)	17 (65)	1 (4)	5 (19)	0 (0)	3 (12)	1 (4)
1–< 2 yr (*N* = 19)	0 (0)	0 (0)	11 (58)	16 (84)	2 (11)	3 (16)	2 (11)	3 (16)	0 (0)
2–10 yr (*N* = 35)	2 (6)	1 (3)	18 (51)	28 (80)	1 (3)	10 (29)	0 (0)	3 (9)	1 (3)
11–20 yr (*N* = 35)	6 (17)	1 (3)	20 (57)	31 (89)	0 (0)	22 (63)	0 (0)	6 (17)	1 (3)
21–29 yr (*N* = 123)	37 (30)	7 (6)	84 (68)	116 (94)	6 (5)	81 (66)	2 (2)	32 (26)	7 (6)
30–45 yr (*N* = 163)	62 (38)	12 (7)	115 (71)	158 (97)	3 (2)	108 (66)	1 (0.6)	37 (23)	11 (7)
> 45 yr (*N* = 114)	46 (40)	6 (5)	74 (65)	111 (97)	5 (4)	75 (66)	0 (0)	19 (17)	7 (6)
**Total** (*N* = 701)	194 (28)	27 (4)	451 (64)	655 (93)	26 (4)	405 (58)	6 (1)	163 (23)	29 (4)

* CMV = cytomegalovirus; HSV = herpes simplex virius.

**Table 2:** One-way analyses of factors associated with *Toxoplasma gondii* seropositivity in 1,857 TORCH-tested patients from Qatar, 2005–2008

Risk factor	IgM positive* (%)	Odds ratio (95% confidence interval)	IgG positive (%)	Odds ratio (95% confidence interval)
Sex				
Female	49 (4)	1.00	378 (33)	1.00
Male	27 (4)	0.91 (0.56–1.46)	194 (28)	**0.79 (0.64–0.97)**‡
Age group				< 0.01†
≤ 6 mo	3 (1)	Undefined	80 (23)	**6.85 (1.63–28.8)**§
7–< 12 mo	0 (0)	Referent	2 (4)	1.00
1–< 2 yr	1 (3)	Undefined	1 (3)	0.68 (0.06–7.77)
2–10 yr	1 (1)	Undefined	6 (8)	2.06 (0.40–10.7)
11–20 yr	3 (3)	Undefined	15 (15)	**4.01 (0.88–18.3)**¶
21–29 yr	27 (6)	Undefined	151 (31)	**10.2 (2.45–42.8)**§
30–45 yr	33 (6)	Undefined	229 (40)	**15.6 (3.75–64.8)**§
> 45 yr	8 (4)	Undefined	87 (46)	**19.4 (4.58–82.3)**§
Nationality‖		0.42†		< 0.01†
Arabian Peninsula	28 (4)	1.00	168 (24)	1.00
African	15 (5)	1.27 (0.67–2.41)	129 (42)	**2.39 (1.79–3.17)**§
American continent	0 (0)	Undefined	6 (32)	1.50 (0.56–4.01)
Asian	19 (3)	0.80 (0.44–1.45)	182 (30)	**1.41 (1.10–1.80)**§
East Medi-terranean/East European	13 (7)	1.73 (0.88–3.41)	79 (40)	**2.16 (1.55–3.02)**§
Other/unknown	1 (6)	1.53 (0.20–12.0)	8 (47)	**2.89 (1.10–7.62)**‡
Year tested		0.23†		0.75†
2005	27 (5)	1.00	160 (32)	1.00
2006	17 (4)	0.78 (0.42–1.45)	119 (30)	0.91 (0.68–1.21)
2007	19 (4)	0.69 (0.38–1.26)	157 (32)	0.97 (0.74–1.26)
2008	13 (3)	**0.50 (0.25–0.98)**‡	136 (29)	0.87 (0.66–1.14)
CMV-IgG seropositivity				
No	4 (4)	1.00	15 (14)	1.00
Yes	72 (4)	1.12 (0.40–3.12)	557 (32)	**2.90 (1.67–5.05)**§
*N* = 1 missing data				
CMV-IgM seropositivity				
No	76 (4)	1.00	550 (31)	1.00
Yes	0 (0)	Undefined	22 (34)	1.15 (0.68–1.95)
*N* = 1 missing data				
Rubella IgG seropositivity				
No	27 (5)	1.00	199 (34)	1.00
Yes	49 (4)	0.84 (0.52–1.36)	373 (29)	**0.82 (0.67–1.01)**¶
HSV-1 IgG seropositivity				
No	20 (3)	1.00	177 (25)	1.00
Yes	56 (5)	**1.73 (1.03–2.90)**‡	395 (34)	**1.53 (1.24–1.89)**§
HSV-1 IgM seropositivity				
No	76 (4)	1.00	566 (31)	1.00
Yes	0 (0)	Undefined	6 (38)	1.35 (0.49–3.74)
HSV-2 IgG seropositivity				
No	62 (4)	1.00	433 (31)	1.00
Yes	14 (3)	0.68 (0.38–1.23)	139 (30)	0.98 (0.78–1.23)
HSV-2 IgM seropositivity				
No	65 (4)	1.00	527 (30)	1.00
Yes	11 (11)	**3.03 (1.55–5.94)**§	45 (43)	**1.74 (1.17–2.60)**§

* Three patients missing IgM results were excluded.

† Global *P* value.

‡ *P* < 0.05

§ *P* < 0.01

¶ 0.05 < *P* < 0.10

‖ Defined in Materials and Methods.

**Table 3:** Multifactorial analysis of factors* associated with IgG *Toxoplasma gondii* seropositivity in 1,857 TORCH-tested patients from Qatar, 2005–2008

Risk factor	Parameter estimate	S.E.	Odds ratio (95% confidence interval)	*P* value
Intercept	−3.9646	0.7671	–	
Age group				< 0.01†
≤ 6 mo	2.0297	0.7427	8.13 (1.90–4.9)	0.01
7–< 12 mo			1.00	
1–< 2 yr	−0.2952	1.2517	0.74 (0.06–8.65)	0.81
2–10 yr	0.7459	0.7367	2.38 (0.45–12.5)	0.31
11–20 yr	1.4148	0.6641	4.62 (0.99–21.5)	0.05
21–29 yr	2.3200	0.6109	11.40 (2.67–48.6)	< 0.01
30–45 yr	2.6904	0.6097	16.50 (3.88–70.2)	< 0.01
> 45 yr	2.9312	0.6212	21.02 (4.85–91.1)	< 0.01
Nationality				< 0.01†
Arabian Peninsula			1.00	
African	0.8341	0.1532	2.30 (1.71–3.11)	< 0.01
Asian	0.2512	0.1317	1.29 (0.99–1.66)	0.06
American Continent	0.1031	0.5270	1.11 (0.39–3.11)	0.85
East Mediterranean/East European	0.8347	0.1794	2.28 (1.60–3.24)	< 0.01
Other/unknown	0.9355	0.5181	2.55 (0.92–7.04)	0.07
CMV IgG+	0.5862	0.3020	1.94 (0.99–3.25)	0.05
HSV-1 IgG+	0.2983	0.1205	1.35 (1.06–1.71)	0.01
Rubella IgG+	−0.5261	0.1205	0.59 (0.47–0.75)	< 0.01

* The table includes only those factors that retained significance after backward selection of the full factorial model.

† Global *P* value.

**Table 4:** Multifactorial analysis of factors* associated with IgM *Toxoplasma gondii* seropositivity in 1,854 TORCH-tested patients from Qatar, 2005–2008

Risk factor	Parameter estimate	S.E.	Odds ratio (95% confidence interval)	*P* value
Intercept	−2.5587	0.2261	–	
Year tested				0.04†
2005			1.00	
2006	−0.3072	0.3190	0.74 (0.39–1.37)	0.34
2007	−0.6420	0.3229	0.53 (0.28–0.99)	0.05
2008	−0.9570	0.3578	0.38 (0.19–0.77)	0.01
HSV-2 IgG+	−0.7200	0.3228	0.49 (0.26–0.92)	0.03

* The table includes only those factors that retained significance after backward selection of the full factorial model.

† Global *P* value.
